# Nosode Prescribing

**Published:** 1893-12

**Authors:** 


### NOSODE PRESCRIBING.

Extract from Proceedings of I. H. A.

### Dr. J. H. Allen's Paper. Miasmatic Diseases.

### Discussion.

Dr. G. H. Clark—The subject of this paper is one in which I have been deeply interested for some years. I am not in harmony with some of Dr. Allen's conclusions. I do not see how he can get out of the corner, that in using these nosodes as he does, he is prescribing on something else than the totality of the symptoms. Every year there is creeping into our meetings more and more of this tendency to base the prescription on something else than the totality, and this way of using nosodes merely on the name of the disease, Syphilinum for syphilis, Medorrhinum for sycosis, Tuberculinum for tuberculosis, and so on, is death to true science. We might as well have no materia medicas. It will drag others into the same fatal error.

Dr. Pease—I would like to ask the members of the society whether it is not very frequent for a psoric, syphilitic, or sycotic patient to experience all the symptoms of a bad cold, while under treatment. I have noticed this a number of times. If this is so, it is a part of the cure, and I should think it unwise to interfere with it. I think I have made a mistake in prescribing for such acute attacks, and in that way interfering with the curative process.

Dr. Hawkes—In the first place, I want to express my hearty approval of what the essayist has said. It confirms remarkably my own experience. The fundamental principle which Dr. Allen expresses, and which has been taken exception to, is a sound one. If we want to prevent small-pox, we would use the vaccine virus. Now, the prophylactic remedy acts as the curative remedy acts, by preparing the system to resist some poison. The totality of the symptoms must be the guide; but there is a great difference in the intrinsic value of different symptoms, and that is where the judgment, experience, and wisdom of the prescriber are shown. It is not a question of number of symptoms entirely; it is rather a question of the relative importance of symptoms. Three important symptoms in a given case may outweigh in value six, or even more trivial ones, and in the question of relative importance, we must consider the miasm, the modalities, and the personal peculiarities of the patient. I think it is a good paper and will lead us aright. In answer to Dr. Pease's question, if I may be permitted to answer it, I think it is a poor plan to interfere with the acute symptoms that arise during the treatment of a chronic case. I teach my classes not to do it, and I never do it myself.

Dr. Pearson—The statement has been made that the difference between a great man and an ordinary one is only one step, one little thing. In the treatment of chronic diseases, the difference between success and failure is just a little thing apparently unimportant—*i. e.*, the knowledge of miasms. This knowledge gives its possessor the advantage, the inside track over the man who does not have it. We should strive in every possible way to differentiate between the various miasms. I think this paper contains some very important hints on a deep and obscure subject which needs much study. We should pay attention to it, and complete it, until it is plain to every practitioner. In regard to these nosodes there is an underlying principle which is very pertinent to the cure of the case. In the first place, nosodes are obtained from diseased persons, human beings like ourselves. It represents characteristic disturbances. When proved upon healthy persons we get the characteristic indications of this remedy. They then become a part of our materia medica, and when given according to the symptoms, I believe they have a more potent effect than any other kind of remedies, owing to their origin.

Dr. Reininger—The question of treating disease by the products of disease is beginning to interest the old school. They have attempted recently to cure diphtheria with a product from the membrane given by hypodermic injection, and have been to a certain extent successful. I noticed one case of psoriasis that had been cured by an old-school physician, by injecting a preparation of the same poison under the skin. I partly agree with Dr. Clark and partly with Dr. Hawkes. Here we have a scarcely trodden field, much experimentation must yet be made, and I doubt not that when we know more about this subject that many stubborn, well-nigh incurable cases will be much more easily handled.

Dr. Wesselhœft—There is only one possible way of getting at this thing, and that is by making provings of the nosodes. These are the only experiments we are called upon to make. I do not believe that any other kind of experimentation is justifiable. As regards the question of Dr. Pease, any interference with the first effect of a remedy in the treatment of an acute disease is always detrimental to the patient. Irritation of the mucous membranes is very apt to follow the administration of the proper remedy in a chronic case, and it should never be interfered with. There is nothing more than a reaction on the internal skin. The nearer the natural orifices it is the better. A nasal irritation is better than one in the trachea, for instance. The same thing is true with irritations of the intestinal tract; you may have them as a constipation or as a diarrhœa. These are natural results of giving a proper remedy, and we make a mistake when we allow the patient to influence us and give them something for the acute trouble which has supervened. What is a cold? People say a cold is a cold, everybody takes cold. A cold is simply a suppression of skin activity, without reaction. Something has got to occur if there is no reaction. Every force that affects the body has got to be accounted for. If we have no surface or skin reaction we are bound to have coryzas, diarrhœa, constipation, or some other effect.

Dr. H. C. Allen—I must take a shot at my friend, Dr. Clark. The one or two nosodes that we have proved were the nosodes used in this paper. Any one can follow their pathogenesis with a great deal of accuracy. In that my friend Clark is off. Take this paper in connection with one read some days ago at a meeting of the American Institute on the surgical treatment of pleurisy.

It was said there that fifty cases of pleurisy, under homœopathic treatment, had required surgical interference. Debate was shut off, so that not much was brought out. One fact that was certain was not brought out, and that was that those cases could not have been treated homœopathically with such wretched results. Another fact should have been exploited, and that was that there is always in acute pleurisy and pneumonia a psoric basis. While Aconite works well when indicated in the early stages, in nine cases out of ten the second remedy is Sulphur and not Bryonia nor Phosphorus. This is what large numbers in our school overlook. The psoric miasm is at the bottom of the majority of acute troubles. When I give a dose of medicine for a chronic disease I never, under any circumstances, disturb an acute trouble that may follow. It is the response to the remedy.

Dr. Butler—Where can we find a good proving of Syphilinum or Medorrhinum?

Dr. H. C. Allen—In Hering's *Guiding Symptoms* you will find Medorrhinum.

Dr. Holmes—Dr. Berridge has also made a proving of Medorrhinum that I think is preferable to Hering's. Dr. Allen's paper is an excellent one. It brings up so many points which no one but those who have made a study of and believe in *The Organon* understand or appreciate. These cases are unique. I believe that nothing but the line of treatment given by Dr. Allen would ever have cured them. They are cases that never get well of themselves—that never get well under any other kind of treatment. They form a part of that vast army of chronics whose lives are hopelessly blighted. The way in which the doctor took those cases from point to point past most dangerous places should command our admiration. If you look at the face of Dr. Allen you will see at once that it is not the face of an enthusiast, he is not a man to be led astray or to lie to or deceive us. Those who never cure such cases are the only ones who raise a question as to the work we are doing here.

Dr. Custis—Dr. H. C. Allen struck the key-note when he spoke of the relation between these remedies and miasms on one hand and acute manifestations of disease on the other. We are about entering upon a season where we will have great numbers of acute diarrhœas to meet, and in these I have found the greatest help from these remedies. But when and how are we to use them? My experience has been that these sudden, severe diarrhœas are not helped in the beginning by nosodes, but when they are not relieved by the indicated remedy promptly as they should be, and the child shows symptoms of marasmus, I have then found that the nosode affects the system so that the symptoms indicating the next remedy come right to the surface. The sequence of remedies is a subject of great importance, and one that needs much study. I have never seen a nosode carry a case through to a cure, but I have many times seen a nosode bring out the symptoms for the next remedy. First comes the remedy which meets the acute symptoms, then the nosode or antipsoric which is most similar to the case, then comes the picture of the remedy, which will cure the case. The manifestation of the action of the remedy, which appears upon the mucous membranes, is a most natural thing when you consider that the mucous membrane is the first surface upon which these miasms impinge. These nosodes become more valuable the more we study them in their connection and sequence with other remedies.

Dr. Hoyne—If we follow the advice of the last speaker I think we will have to do a great deal of guessing. I believe we must cover the symptoms, whether the remedy is a nosode or not, and this rule would shut out all nosodes that are not proved. We cannot have a case of pleurisy without effusion. It is a part of the natural history of the disease, whether the patient is psoric or not, and hence it is not a proof of a psoric constitution. Certain remedies, not necessarily anti-psoric, have this condition of pleura—*i. e.*, congestion followed by effusion—among their symptoms. They are the remedies usually from which we make our choice of one which fits all the peculiarities of the case in hand. Now, as I understand the speaker, we are to select a remedy for the acute attack, then give a nosode which will develop symptoms, then another remedy for those symptoms. That may be a good method. I have never tried it, but it is not Homœopathy. Why give a nosode without symptoms for it? If the first remedy is selected correctly you may need a second remedy, which possibly may be a nosode, but it is not right to select a nosode because it is a nosode. It is my experience that nosodes are seldom indicated in acute affections. I have run a skin and venereal clinic for many years, and have had a large experience in gonorrhœa, and I do not think that Medorrhinum is indicated in one case out of a thousand. It seems to me that Dr. Custis' remarks were confusing, and not homœopathic.

Dr. Custis—If my advice is followed, guessing is what we would not have to do. What is the use of practicing ten or fifteen years, if we do not accumulate experience? Now, it is the result of experience that on a good, honest constitution acute diseases do not act as they do on scrofulous or psoric bases. They do not leave bad results. Bad results in the shape of chronic diseases occur only in constitutions laboring under miasms. We have a right, and it is our duty to anticipate these conditions, which experience has taught us are almost sure to come. It is not unhomœopathic that I can see. Without the benefit of such experience you will relieve your patient of the acute trouble he may have, but you will not make a complete cure until you have well studied the anti-psoric. At the same time the anti-psorics, and especially the nosodes, are dangerous remedies, and must be used with great care. We must know the right dose and the right repetition.

Dr. Clark—I am thoroughly at one with Dr. Holmes in attributing honesty of purpose to Dr. Allen, but I cannot agree with him that he is correct in his treatment of miasms. Boiling it right down you have nothing but this: you assume that a given case of sickness is due to sycosis, or syphilis, or to tuberculosis, and then on this assumption you give Medorrhinum for sycosis, Syphilinum for syphilis, Tuberculinum for tuberculosis, and so on. You are getting right back to the old school error of treating names of diseases instead of individuals. I

never received a greater shock in my life than when I found out that Dr. Swan knew literally nothing about the history or origin of his nosodes. His Medorrhinum is taken from a case of gonorrhœa, of whose history we know absolutely nothing. Now, does any one, for a moment, claim that one case of gonorrhœa will represent all cases ?

Dr. Lippe insisted that we must prove the nosodes or we would fall into mere routine prescribing by name. There was a man in our school who knew how to use these nosodes. That man was the late Dr. Fellger, of Philadelphia.

He insisted that we should know as much as possible about the individuals from whom the preparation was taken. For instance, he had a preparation of Variolinum taken from a case of the confluent variety with certain peculiarities of eruption, and of a certain temperament and constitution. That was the kind of a man to depend on when it comes to nosodes.

Dr. Hawkes—It seems to me that that is an over-refinement. We do not select vaccine from a black heifer for one kind of patient and from a red heifer for another kind. We simply get the vaccine virus from a cow, and it works. No one can question the fact that vaccine and vaccination have been universal modifiers of small-pox. The prophylactic remedy acts precisely as the curative remedy acts, by making the patient capable of resisting the assault of some miasm. Pettenkofer has demonstrated that a healthy man can swallow countless millions of cholera microbes without getting the cholera. A remedy, whether prophylactic or curative, puts a man into a state which enables him to stand the attack of some disease-producing influence. The history of small-pox and vaccination is sufficient to support the position the essayist took, and that I took when I spoke of the value of symptoms. The similarity between the nosode and its origin to the diseased condition is sufficient to make that one fact of great weight in its selection for a case. Intelligent and conscientious men and women would not disagree nearly as much as they do if they could decide on the exact meaning of the terms they use. Dr. Hoyne's remarks brought this to mind. The meaning of the phrase " totality of symptoms" is different in different minds. Properly the totality of the symptoms includes the history of the patients and of his father and mother. It includes every part of heredity. That being the case, then these symptoms, whether acquired, or hereditary, or miasmatic, have weight and must be studied. Some are more valuable than others, some are less valuable.

Dr. Clark—Then you believe in *tollere causam?*

Dr. Hawkes—Certainly; the cure of disease is altogether *tollere causam*, but you must know what the cause is, and you will find it among the miasms.

Dr. Waddell—As I understood Dr. Allen, he did not recommend such a crude procedure as to prescribe Medorrhinum for gonorrhœa, Syphilinum for syphilis. It was, in effect, to consider a history of syphilis, or of gonorrhœa as important elements in the case, and as having a bearing on the prescription. In one case he prescribed Mercury; in another, Syphilinum, and so on, simply because the symptoms indicated these remedies.

Dr. Pease—I believe that most of our failures in the cure of chronic cases arise from the fact that our patients come to us after a long course of drugging; they come filled up with hypnotics, bromides, quinine, the antipyretics, and what not. Before we can really begin to get at the reaction, I think it is necessary to antidote as far as possible these drug symptoms. Where we can find out the drug last taken, we can antidote it by giving the same drug in a high potency, as Dr. Allen, in his paper, antidoted the effects of Mercury by giving Mercury high. I know that Dr. Sawyer, in a great many cases in which he is successful, follows this plan. I have also found it a good plan in my own experience.

Dr. A. R. Morgan—It seems to me if these methods of treatment are legitimate, there is very little use of our studying medicine. If Isopathy is to precede Homœopathy what is the use of studying materia medica? I have little experience with any nosode except Psorinum, and I think from what I have here heard that we are drifting toward a dangerous error. When I come across a tedious or difficult chronic case I do not resort to Isopathy. I fall back on the psoric miasm and select an anti-psoric remedy. Oftentimes a dose of Sulphur will revive the latent susceptibility of the patient. I do not object to the use of any nosode that has been proven and that is prescribed on the symptoms.

Dr. Butler—I also want to protest against nosodopathy. It is not Homœopathy, it is not even Isopathy. A nosode is only one of the many products of disease, and may or may not be a powerful remedy. But I want to emphasize the fact that without good provings we are at sea. I do not believe there exists a good proving of Medorrhinum or of Syphilinum. What we have are made by men of extreme credulity. I do not want to speak of Dr. Swan with any disrespect, but still I must say that Dr. Swan is the most credulous man I ever had any dealings with. You will find symptoms of gonorrhœa put down as belonging to the pathogenesis of Medorrhinum. I have little faith in the provings of most of our nosodes. The proving of Psorinum dates back many years, and is no doubt an excellent one. It has been extensively verified. In regard to Isopathy my experience with it is not favorable. I have not had good results from giving Mercury high, for chronic mercurial poisoning for instance.

Dr. H. C. Allen—Hering was the prover of Lyssin.

Dr. Butler—Hering was an excellent man. He was probably the best man that ever lived for taking clinical cases and selecting the truly curative drug to be given, but Hering was human.

Dr. Clark—Hering himself says there is no proving of Lyssin. What we have was taken from the effects of bites of rabid dogs.

Dr. J. H. Allen—We all believe in the law of similia. We all believe that no cure can be effected without the application of similia. Our differences, I think, arise from misunderstanding. I have no use for name-prescribing any more than the rest of you. I have prescribed Medorrhinum a thousand times, I suppose, without any result at all, and yet I have unshaken faith in the proving of Medorrhinum as far as it goes, for when it is the simillimum it will cure your case. No matter what the agent is, so you adhere to the law it will cure. The first case I reported had been treated by a homœopathic physician for five years. I treated it for four years, and finally decided that Medorrhinum was the simillimum, and gave it. It was very far from prescribing on the name of the disease. You can see what nerve it took to wait three or four months on a single dose. Medorrhinum nearly cost him his life. The second case I had nine months for syphilitic eruption. I was called in to see the child's mother in puerperal convulsions. There was a history of syphilis on both sides of the house.

It is not yet possible to get a complete proving of these remedies. Who is going to prove them? Must we wait and not use them on that account when we have enough to do valuable work with? We all use Calc-carb., and yet the most valuable symptoms of Calc-carb. are clinical or pathological, and we prescribe it on those symptoms lots of times. Also Sulphur as an inter-current.

I have found that where a chronic case is covered up by a suppression that the nosodes most similar to the miasm at work in the patient that it will take the case to a point where you can see the similar remedy.

Bureau closed.

